# On-Line Flatness Measurement in the Steelmaking Industry

**DOI:** 10.3390/s130810245

**Published:** 2013-08-09

**Authors:** Julio Molleda, Rubén Usamentiaga, Daniel F. Garcίa

**Affiliations:** Department of Computer Science and Engineering, University of Oviedo, E-33204 Gijon, Spain; E-Mails: rusamentiaga@uniovi.es (R.U.); dfgarcia@uniovi.es (D.F.G.)

**Keywords:** shape measurement, flatness measurement, manifest and latent flatness, mechanical flatness sensor, optical flatness sensor, tensile stress measurement, optical triangulation, surface reconstruction

## Abstract

Shape is a key characteristic to determine the quality of outgoing flat-rolled products in the steel industry. It is greatly influenced by flatness, a feature to describe how the surface of a rolled product approaches a plane. Flatness is of the utmost importance in steelmaking, since it is used by most downstream processes and customers for the acceptance or rejection of rolled products. Flatness sensors compute flatness measurements based on comparing the length of several longitudinal fibers of the surface of the product under inspection. Two main different approaches are commonly used. On the one hand, most mechanical sensors measure the tensile stress across the width of the rolled product, while manufacturing and estimating the fiber lengths from this stress. On the other hand, optical sensors measure the length of the fibers by means of light patterns projected onto the product surface. In this paper, we review the techniques and the main sensors used in the steelmaking industry to measure and quantify flatness defects in steel plates, sheets and strips. Most of these techniques and sensors can be used in other industries involving rolling mills or continuous production lines, such as aluminum, copper and paper, to name a few. Encompassed in the special issue, *State-of-the-Art Sensors Technology in Spain 2013*, this paper also reviews the most important flatness sensors designed and developed for the steelmaking industry in Spain.

## 1. Introduction

In the steelmaking industry, shape is a key characteristic to determine the quality of outgoing products of rolling mills and downstream manufacturing lines. This characteristic is essential in modern high-speed and high-volume finishing lines, where flat-rolled products must fulfill tight shape tolerances. Furthermore, shape is a main feature used by customers to accept or reject incoming material provided by steel suppliers. Thus, new operating strategies aiming to reduce shape defects and yield losses are continuously being proposed [1,2] for steel rolling and processing. The shape of flat-rolled products is greatly influenced by flatness. In rolling mills, flatness measurement and control are critical success factors, which improve the quality of the manufactured products, as well as minimizing rejects, processing time and breaks during manufacturing. Besides improving the quality of the manufactured product, measuring and controlling flatness also increase the productivity of the steelmaking plant, since products with poor flatness may cause great vibrations and move imprecisely, or even break, in downstream processes.

From a mathematical point of view, flatness can be defined as the degree to which all the elements of a surface approach a plane [3]. In flat-rolled products, flatness defects are a consequence of roller thermo-elastic deformation, which affects the roller gap in the roller stand and results in heterogeneous rolled-product plastic deformation. These deformations are related to residual tensile and compressive strains, or residual stress, across the product width and thickness [4]. Flatness defects in flat-rolled products are generated due to non-uniform distribution of rolling pressure in the rolling stands, which leads to out-of-bite stress gradients, resulting in buckles in the compressive area. The origin of stress gradients lies in the rolling method—symmetric or asymmetric—and the difference between the thickness profile of the incoming workpiece and the roller gap. This thickness profile is called crown. Residual stress across the product width provokes longitudinal waves, whereas residual stress across the product thickness generates cross bow. Flatness defects can also be generated due to roll thermal crown and uneven temperature during cooling, leading to uneven contraction of the rolled product. Depending on the stress profile, waves on flat-rolled products can be observed in all directions: longitudinal, transverse or oblique.

Modern rolling mills combine higher rolling speeds, larger reductions of the incoming workpiece, harder steel grades and thinner rolled output products. Thus, to ensure high quality levels in terms of flatness and defect-free surfaces, knowledge of friction and lubrication in the roller gap becomes critical [5]. Some sensors have been developed to measure the stress in the roller gap [6]; however, these sensors may mark the surface of the rolled product. New indirect friction sensors are proposed to avoid this issue [7] and use inverse analysis to compute the friction of the roller and the product. Inverse methods can also be used to measure contact stress and temperature in the roller gap; among them, analytical approaches provide higher accuracy [5]. The output provided by flatness sensors after rolling allows these methods to trace the sources of flatness defects in the roller gap.

**Figure 1.**
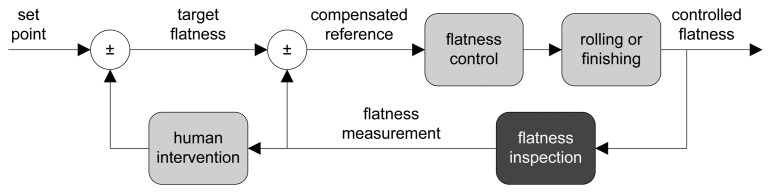
Feedback block diagram of a flatness control system.

Usually, flatness tolerances are determined depending on the use of the manufactured product and on the requirements imposed by the customer. To achieve the desired flatness, a flatness control system—which drives the mechanical and thermal actuators to adjust the shape of the product—is used. Mechanical actuators adapt the roller gap, the bending and the tilting of each rolling mill stand [8], whereas thermal actuators try to achieve a uniform temperature of the product while cooling [9]. Usually, a flatness control system is a closed-loop controller, which uses the feedback provided by a flatness sensor measuring the flatness after rolling to compensate for the original set point [10], as shown in Figure 1. Typically, flatness sensors compute a surface topography of the rolled product; then, geometrical measurements are derived, and thus, flatness measurements can be taken.

The improvement of shape quality in the steelmaking industry began in the early 1960s with the improvement of width and thickness accuracy. In those days, flatness measurements were performed off-line and manually by humans, until the first automated devices were developed in the late 1960s. The first automated flatness sensor for steel rolling mills was delivered in 1967, as ABB (prior ASEA)claims. Early automated flatness sensors were contact devices based on mechanical principles. They used strength or pressure transducers integrated into a deflector roller to measure the radial force applied by the product as it moved along the manufacturing line. The principle of measurement was based on the fact that radial force along the rolling direction is directly proportional to the tension profile in the rolled product, that is, product tension plus residual stress, assuming the shear stress is negligible. In the mid-1980s, non-contact flatness sensors based on optical principles started to be developed. They mainly used triangulation and structured light to compute the 3D surface of the product under inspection. Both mechanical and optical principles are currently used in modern flatness sensors for the steelmaking industry. The main advantage of mechanical systems is that they can be installed in very harsh atmospheres, where optical systems will be likely to fail. However, they cannot be used with very thick or very hot rolled products, because the pressure sensors might be damaged. Conversely, optical flatness sensors provide the advantage of non-contact sensors for high quality surface manufacturing. In the steelmaking industry, high quality surfaces are referred to as technically free from manufacturing cracks [11,12]. Although optical sensors were more expensive, the development of machine vision devices at decreased cost, as well as their miniaturization and integration in industrial processes, have accelerated the use of this type of sensors in industry [13]. Flatness sensors in the steelmaking industry constitute an example of such integration.

Modern automated flatness sensors in the steelmaking industry are required to provide on-line flatness measurements. First, these measurements are required as feedback for flatness control systems (see Figure 1). Second, real-time monitoring of product conditions is required in every modern steel rolling mill and processing line. On-line flatness measurement is becoming more and more difficult in current manufacturing lines, where processing speed is ever increasing, and thus, on-line sensors need to meet very narrow deadlines. Furthermore, the high quality requirements imperative to manufactured products in modern steelmaking lines impose high levels of accuracy on these flatness sensors. In addition to feedback for flatness control systems and real-time monitoring, flatness measurements are used by steel suppliers to classify their outgoing products and by customers to assess the quality of the incoming material. Sometimes, these tasks rely on standards that define the maximum tolerances for each product type [14,15].

This paper reviews the technologies and applications used for on-line flatness measurement in the steelmaking industry to measure and quantify flatness defects in steel plates, sheets and strips. Most of them can be used in other industries where rolling mills or continuous production lines are used, such as aluminum, copper and paper. The rest of this paper is organized as follows. Section 2 describes how flatness defects are generated in steel, flat-rolled products in rolling mills and production lines, the main types of flatness defects and how they can be quantitatively evaluated. Section 3 describes the main techniques used in on-line flatness measurement systems in the steelmaking industry and the main flatness sensors developed based on each technique that are commercially available. Since this paper is encompassed in the special issue, *State-of-the-Art Sensors Technology in Spain 2013*, Section 4 reviews the most important flatness measurement sensors designed and developed for the steelmaking industry in Spain in the last three decades. Finally, Section 5 shows a comparison of the main features of all the flatness sensors described in Sections 3 and 4, as well as the final impressions of this review.

## 2. Flatness of Steel, Flat-Rolled Products

Steel, flat-rolled products—such as plates, sheets and strips—are manufactured from large, hot slabs that are passed through several rolling stands in a rolling mill, reducing their thickness and increasing their length. The rolling process can be symmetric or asymmetric. Symmetric rolling applies equal rolling conditions at the roll-slab interface by keeping the roller diameters, the roller speeds, the roller-slab friction and the slab entry between rollers symmetrical with respect to the middle plane of the slab. The output of symmetric rolling is straight, flat-rolled products. On the other hand, asymmetric rolling can arise for several reasons, such as difference of the surface roughness or diameters of the rollers or when rolling bimetallic slabs. The output of asymmetric rolling is curly, flat-rolled products [16].

In the steel industry, it is well known that when the thickness profile across the width of the slab is constant, the outgoing flat-rolled product will have non-flat edges, since the edges of the slab can expand to the free space of the roller gap. To compensate for this effect, the rollers in the rolling stands are bent to laminate the slab with a predefined thickness profile, which maximizes the likelihood of the rolled product being perfectly flat [8]. In addition, the rollers can be tilted as a result of the analysis of the flatness feedback received to improve the final flatness. An incorrect estimation of the bending and the tilting parameters of the rollers may lead to flatness defects in the rolled products. In the steel industry, it is well known that when the thickness profile across the width of the slab is constant, the outgoing flat-rolled product will have non-flat edges, since the edges of the slab can expand to the free space of the roller gap. Once the slab is rolled, the outgoing flat product is cooled. The evolution of the temperature distribution of the rolled product during cooling is also of utmost importance to avoid flatness defects [9]. If the rolled product is cooled very fast, residual stresses may cause non-flat edges [17]. Furthermore, the flatness of the rolled products may be affected in other manufacturing stages, such as coiling, cutting and lifting, among others.

**Figure 2.**
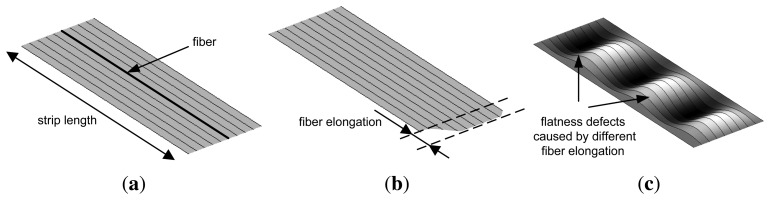
Flat-rolled product considered as multiple, adjacent longitudinal fibers: (**a**) rolled product after rolling with all fibers featuring the same length; (**b**) theoretical model of a product showing different fiber length; (**c**) realistic model of a rolled product after rolling showing flatness defects, due to different fiber elongation.

A quantitative evaluation of flatness defects in flat-rolled products is commonly carried out considering the rolled product divided as multiple, adjacent longitudinal fibers, as shown in Figure 2. When all the fibers of a rolled product have the same length after rolling, the product is considered perfectly flat, as shown in Figure 2a. Rolled products featuring the same length for all the fibers may have longitudinal undulations. In this case, all the points of the product surface do not belong to the same plane, but in the steelmaking industry, this product is considered flat, since it is very easy to remove these undulations (in a skin-pass stage, for instance). If each longitudinal fiber of the product could be elongated independently in the rolling process, the final product would appear as shown in Figure 2b. However, in a real rolled product, the elongation of different sections affects adjacent sections, due to internal tensions, and a rolled product with different fiber elongations would feature waves, as shown in Figure 2c and, thus, flatness defects.

**Figure 3.**
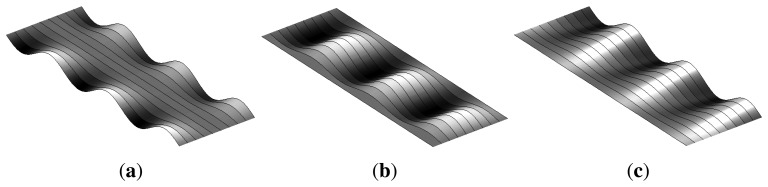
Common flatness defects of steel, flat-rolled products: (**a**) wavy edges (fibers at the edges are longer than fibers at the center); (**b**) center buckle (fibers at the center are longer than fibers at the edges); (**c**) bad leveling (the length of the fibers increase from one edge to the other).

Waves in flat-rolled products may appear in different sections, depending on the location of the fiber elongations. When the length of the fibers at the edges of the rolled product is longer than the length of the fibers at the center, the rolled product is said to have a flatness defect, called wavy edges, as shown in Figure 3a. Conversely, when the length of the fibers at the center is longer than the length of the fibers at the edges, the product features a flatness defect, called the center buckle, as shown in Figure 3b. Another flatness defect appears when the length of the fibers increases from one edge of the rolled product to the other. This defect is called bad leveling and is shown in Figure 3c. These are three major flatness defects that may appear when residual stress distribution is homogeneous across the product thickness and heterogeneous across the product width, mainly in symmetric rolling. In asymmetric rolling, heterogeneous stress distribution across the product thickness may also appear, generating cross bow flatness defects.

Flat-rolled products in the steel industry are considerably long, especially strips that can be several hundreds of meters long. Thus, longitudinal sections of the rolled product having a reference length, *L_ref_*, are used to report its flatness. Typically, *L_ref_* takes values from 2.5 to 10 meters.

Usually, flatness defects in the steelmaking industry are quantitatively evaluated using a dimensionless metric, called I-units [18], computed as shown in Equation (1), where *F_j_* is the I-unit index of the fiber, *j*, and Δ*L_j_*/*L_ref_* is the elongation of the fiber, *j*, compared to the reference fiber, *ref*. The factor used by the I-unit index (10^5^) made it equivalent to 10 *μ*m/m.

(1) $$F_{j}=\frac{\Delta L_{j}}{L_{\text{ref}}}10^{5}=\frac{L_{j}-L_{\text{ref}}}{L_{\text{ref}}}10^{5}$$

As shown above, flatness measurements of a rolled product in the steelmaking industry can be computed based on length measurements taken over several longitudinal sections of the product. However, there are some factors that may make these length measurements inaccurate in some manufacturing processes: the tension of the product, fast tension variations, the deflection angle, the temperature profile and the product thickness. When flat-rolled products are processed with no tension, or under low tension levels, the waves of the product are not modified, and the flatness defects can be directly seen. In this case, flatness defects are said to be manifest. Conversely, when the products are processed under high tension levels, this tension may mask some flatness defects or the real magnitude of these defects. In this case, flatness defects are latent, that is, they cannot be seen directly, but they will appear in the product when the manufacturing tension is released. The term, critical buckling stress, is often used to refer to the stress level where latent defects turn into manifest defects. Therefore, when the products are processed under high tension levels, a comparison between the tensile stress of the fibers is preferred to compute their lengths. The flatness index of a fiber of the product can be computed from the tensile stress using the derived formula of Hooke's law shown in Equation (2), where *F_j_* is the I-unit index of the fiber, *j*, Δ*L_j_*/*L_ref_* is the elongation of the fiber, *j*, compared to the reference fiber, *ref*,*σ_j_* is the longitudinal tensile stress of the fiber, *j*,*σ_m_* is the mean longitudinal stress across the product width and *E* is the modulus of elasticity of the steel product.

(2) $$F_{j}=\frac{\Delta L_{j}}{L_{\text{ref}}}10^{5}=\frac{\Delta \sigma }{E}10^{5}=\frac{\sigma_{j}-\sigma_{m}}{E}10^{5}$$

**Figure 4.**
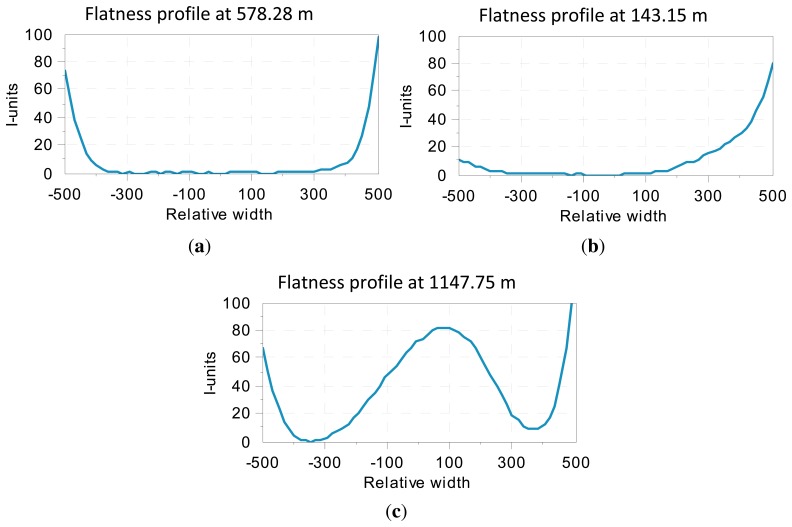
Typical flatness measurements provided by a flatness sensor of a flat-rolled product: (**a**) flatness profile showing wavy edges defect; (**b**) flatness profile showing bad leveling; (**c**) flatness profile showing center buckle and wavy edges. These flatness measurements have been taken by the flatness sensor described in [19].

**Figure 5.**
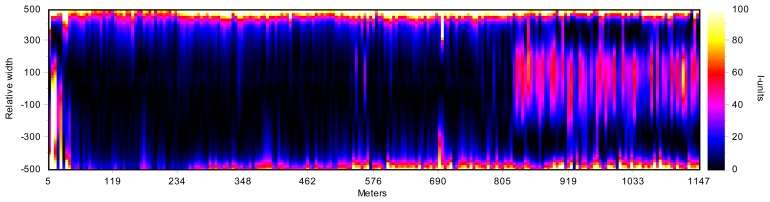
Flatness map built up with the flatness profiles computed for a steel strip. The first section of the product shows bad leveling; the second, wavy edges; and the third, wavy edges combined with center buckle. This flatness map has been computed by the flatness sensor described in [19].

Since the I-unit index expresses the relation between the length of a fiber compared to a reference fiber, a flatness profile can be obtained by computing the I-unit index for each fiber, that is, the distribution of the relative elongation of the fibers. Figure 4 shows three examples of flatness profiles. As mentioned above, the length of flat-rolled products in the steelmaking industry could be up to several hundreds of meters long. Therefore, flatness profiles are usually concatenated, representing a 2D map, called a flatness map, of the whole rolled product, as shown in Figure 5. The flatness profiles shown in Figure 4 are contained in this map. As can be seen in this flatness map, the measured rolled product shows bad leveling in its first 250 meters, with wavy edge defects—with increasing intensity in one of the edges—between meters 250 and 820 and wavy edges combined with center buckle in the last 300 meters.

Once flatness measurements have been computed for all the fibers of the rolled product, two more specific measurements can be provided from them, symmetrical and asymmetrical flatness. Symmetrical flatness measurements can be used to describe flatness defects caused by bending irregularities in the rolling stand and can be computed as shown in Equation (3), where *F*_1+_*_n_*_-_*_c_* is the symmetrical flatness and *L*_1_, *L_c_* and *L_n_* are the length of the fiber at one side, at the center and at the other side of the product, respectively. On the other hand, asymmetrical flatness measurements can be used to detect irregularities in the tilting of the rollers and can be computed as shown in Equation (4), where *F*_1-_*_n_* is the asymmetrical flatness.

(3) $$F_{1+n-c}=\frac{\frac{L_{1}+L_{n}}{2}-L_{c}}{L_{c}}10^{5}$$

(4) $$F_{1-n}=\frac{L_{1}-L_{n}}{\frac{L_{1}+L_{n}}{2}}10^{5}$$

Sometimes, comparisons between the flatness measurements provided by an on-line flatness sensor and the flatness measurements computed using off-line methods of the same rolled product are required. However, it is very difficult to take measurements of the length of several longitudinal fibers of the rolled product off-line. Furthermore, it is not possible to measure the tensile stress across the width of the product, since no tension is applied. Therefore, off-line flatness measurements are usually carried out assuming sine-shaped waves of the rolled product, and after measuring their wavelength and amplitude, flatness measurements are computed using Equation (5), where *H_j_* is the peak-to-peak wave amplitude of the fiber, *j*, and *L_j_* is the wavelength of the fiber, *j*. In practice, on-line measurements recorded under tension cannot always be correlated with the values measured off-line with the aid of other instruments [20]. After being measured on-line, the flatness of the rolled products is further influenced by coiling processes, temperature changes and product deflection in downstream processes. It is very difficult to determine the exact effects of the variation in tension and temperature, since these effects depend on the dimensions and the chemical composition of each rolled product. However, most of the steelmaking plants assume that a general figure of 0.5 I-units is masked per each N/mm^2^ of tension applied by the production line and one I-unit per °C of temperature variation of the rolled product. Thus, flatness measurements taken in two different locations of the steelmaking plant may vary, and such correlation could be very hard to find.

(5) $$F_{j}={\left(\frac{\pi H_{j}}{2L_{j}}\right)}^{2}10^{5}$$

## 3. On-Line Flatness Sensors in the Steelmaking Industry

Usually, flatness sensors provide flatness measurements based on a two-stage operation. In the first stage, topographical information about the surface of the product under inspection is obtained. This topographical information consists of the length of several longitudinal sections, or fibers, of the rolled product. In the second stage, flatness measurements are computed from this information. Typically, flatness sensors are classified based on the technique used in the first stage. Two main criteria can be used: (*i*) whether or not the sensor is required to be in contact with the surface of the product under inspection; and (*ii*) the physical principle used by the sensor. Using the first criterion, flatness sensors are classified into contact and non-contact devices, whereas using the second criterion, they are classified into mechanical or optical devices. In this paper, the first criterion is used, since it is the most commonly used in the steelmaking industry.

### 3.1. Contact Flatness Sensors

Contact flatness sensors are designed to compute the length of longitudinal fibers of rolled products based on the strength across their width when they move forward along the rolling mill or the production line. The pressure patterns measured are used to determine the tensile stress across the width of the rolled product. Then, the flatness index of each fiber can be computed by means of Equation (2). Since contact flatness sensors measure the tensile stress of the rolled product, they are capable of determining latent flatness defects. Flatness sensing based on pressure measurement is widely used today in the steelmaking industry. In fact, it is the most commonly used technique for flatness measurement in cold rolling mills.

**Figure 6.**
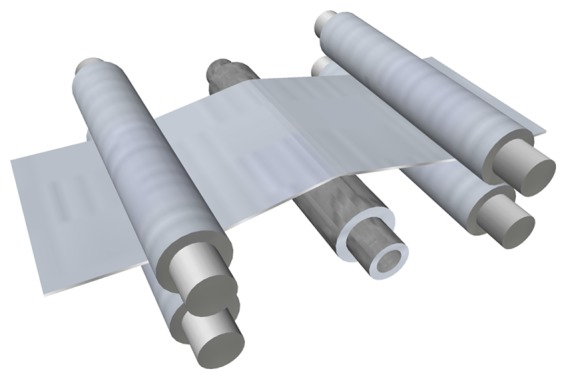
Roll-based flatness sensor (center) installed with a slight deflection of the flat-rolled product to ensure that radial force is generated, while the product is being manufactured.

Contact flatness sensors for flat-rolling mills and production lines are rollers with built-in sensors. These rollers, often called stress-meter rollers, are installed where a slight deflection of the rolled product is caused to ensure that radial force is measured when tension is applied during manufacturing, as shown in Figure 6. This deflection may affect flatness measurement, and thus, models to compensate the roller deflection can be used [21]. Since these sensors are used as deflector rollers, no additional space is required in the production line to install the system. If the mill or the production line was not designed with a built-in flatness sensor, an existing deflector roller can be substituted by a roll-based flatness sensor.

In early roll-based flatness measurement systems, the strength sensors were in contact with the rolled product through holes in the outer layer of the roller [22]. The product under inspection was in contact with the sensors, and thus, patterned marks or scratching could appear on the surface. Another design was based on a roller divided into several discs, also called rings or slices [23,24]. These discs were moved from the main axis of the roll, due to the pressure applied by the rolled product; their movement was captured by pressure sensors. These sensors provided analogue output signals based on their deformation for determining the local internal tension of the rolled product, from which flatness measurements were derived. Individual discs had great form-retaining rigidity and did not create excessively high inertia moment for the measurement system as a whole. In any case, these systems could not be used when products with high quality surfaces were manufactured. Most modern roll-based flatness measurement systems are seamlessly designed [25–27], that is, there is no gap between the strength sensors and the roller surface, or the roller has an extra layer covering these sensors. Although the surface is continuous, measurements are similar to the ones provided by the sliced rollers mentioned above. Thus, roller surfaces are fully homogeneous, and they do not contain mobile parts. These modern sensors can be used where the quality of the product surface is of particular importance. Furthermore, manufacturers may provide the rollers with a wide range of wear-resistant coatings, such as tungsten carbide or chromium plated, which greatly extends their operational life.

The first commercially available, roll-based flatness measurement system Stressometer^®^, was developed by ASEA (currently ABB) in 1967 [28]. It was based on a metallurgical phenomenon according to which mechanical forces alter the ability of some steels to convey a magnetic field, that is, the magnetic properties of a ferro-magnetic material are influenced by the mechanical forces acting on it. In this flatness sensor, an electromagnetic field is created by a row of transducers located close to the surface across the width of the roll. Each transducer, patented as Pressductor^®^ in 1954 [29], detects this force based on the magneto-elastic effect. When there is no external pressure in the roll, no magnetic coupling appears among the transducers. However, when the transducer is subjected to a mechanical force—due to the external presence of a rolled product—the magnetic field pattern is altered, and a voltage level is induced in the transducers. This level is proportional to the force exerted by the rolled product. In current implementations, four rows of transducers are included inside the roll, and thus, four measurements can be taken per roller revolution.

The second well-known technology for roll-based flatness measurement systems was developed by VDEh-Betriebsforschungsinstitut (BFI) [30]. This technology was licensed to several companies that built flatness sensors according to it. In this case, radial force measurement was carried out by piezoelectric sensors [31]. In modern implementations, the piezoelectric sensors are active quartz crystals that emit a voltage level directly proportional to the mechanical load applied. Therefore, a power supply is not required, unlike the ABB system. Thus, the amount of cabling and the complexity of the system is reduced. In the latest designs, sensors are mounted in up to six axially parallel bores. Thus, six measurements per roller revolution can be provided.

Roll-based flatness measurement sensors provide accurate measurements that are consistent when the tension applied to the rolled product varies in the manufacturing process. The main drawback of these systems is that they are intrusive measurement devices, and thus, although they can use very well-treated surfaces, they may scratch or stretch the surface of the product under inspection. Furthermore, contact flatness sensors cannot be used to measure the flatness of steel plates, since they are not in tension during manufacturing. They move along the processing line, due to the effect of rolling stands or motorized rollers underneath them. In all these scenarios, non-contact flatness sensors are preferred.

Usually, roll-based flatness sensors are not suitable for hot rolling mills. The high temperatures of the rolled product may cause rapid wear and tear of the roller, reducing its operational life sharply and increasing the maintenance time and the amount of spare devices required for the production line. A roll-based flatness sensor for flat-rolled products at high temperatures has been patented by Clecim (currently Siemens VAI Metals Technologies SAS) [32]. The roller is cooled down by circulating a heat-exchanging fluid along one portion of the free sector of its external face. The cooling process can be done by immersing one portion of the roller in the fluid or by spraying the roller with it. In any case, the fluid temperature and flow rate are calculated—in relation to the temperature of the product, the speed of the product movement and the thermal exchange conditions—so that the temperature of the external face of the roller is brought back to a pre-set equilibrium temperature at each revolution.

Roll-based flatness sensors are not suitable either to measure thick rolled products (above one or 2 mm). In these products, the critical buckling stress is quite high, and the tension distribution measured with a roll-based sensor would provoke too high of a contact stress between the roller and the product. Furthermore, the deflection angle would provoke plastic deformation in thick products when the product wraps on the roller. Thus, these manifest flatness defects must be measured using another technique. On the other hand, the critical buckling stress is low in thin-rolled products, and any small heterogeneity of internal stress can be masked by the tension in the manufacturing line. Thus, roll-based flatness sensors are used in this scenario to measure the latent flatness defects.

As mentioned above, roll-based sensors are the most commonly used devices for flatness measurement in cold rolling in the steelmaking industry. This type of sensor provides flatness measurements that match with predictions provided by modern numerical simulations [33]. However, roll-based sensors need to overcome one major issue. Since individual sensors are separated by several centimeters in the roller, the accuracy at the edges of the product is not as high as the accuracy provided by modern non-contact optical sensors, as described below.

### 3.2. Non-Contact, Optical Flatness Sensors

Optical methods are widely used for contactless shape measurement in industry to speed up and ensure product development and manufacturing quality [34]. Surface measurements, obtained as depth information by optical methods, are stored as a collection of distance measurements from a known reference coordinate system to target surface points [35]. In machine vision, optical methods are classified as passive and active [36,37]. Passive methods include stereo-vision, shape from shading, shape from motion and shape from texture. On the other hand, active methods include techniques, such as triangulation, moiré topography and holographic interferometry.

Optical sensors in industry tend to use controlled lighting methods, that is, active methods, when they are installed in hostile environments to avoid external noise. The most commonly used method for contactless flatness measurement in the steelmaking industry is active triangulation. Active optical triangulation systems require a controlled light source, which projects a light pattern onto the surface of the product. Flatness defects provoke variations in the surface of the product, and thus, variations in the projected pattern. These variations are imaged by a sensor, typically a charge-coupled device (CCD) camera, and then they are processed to compute the lengths of the fibers of the surface. Although white-light projectors can be used, laser projectors are usually preferred as the active light sources for these systems, given their high spectral radiance, which allows one to reduce the sensor exposure time per measurement.

Since optical flatness sensors are non-contact devices, they cannot measure the tensile stress across the width of the rolled product while manufacturing. Thus, optical flatness sensors are only able to measure manifest flatness defects, as opposed to roll-based flatness sensors that can measure latent flatness defects. Therefore, optical-based flatness sensors require low tension to provide accurate measurements. Furthermore, some optical flatness sensors—mainly those based on active triangulation—may not be suitable for products featuring very bright surfaces.

#### 3.2.1. Laser Scanning

**Figure 7.**
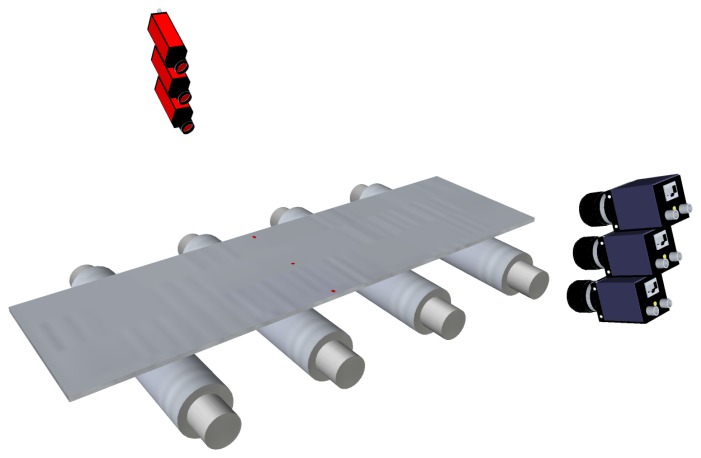
Flatness sensor based on active optical triangulation. Three discrete laser points are projected onto the product surface and imaged using linear cameras. Cameras and laser projectors may be installed on motorized stands or combined with sets of mirrors to track lateral displacements of the rolled product while manufacturing.

Primitive flatness sensors based on active optical triangulation used discrete light points. The points were projected onto the surface of the product to be inspected, and their projections were acquired by linear-sensor cameras, as shown in Figure 7. The movement of these points when the product is being manufactured was used to reconstruct some longitudinal fibers—the same number of fibers as laser points—and then, flatness measurements were computed. Usually, flatness sensors based on discrete points need mechanical devices to track the lateral displacements of the product while moving along the production line—to ensure that the same longitudinal fiber is sampled despite horizontal movements of the product. These mechanical devices were also used to relocate the light spots equidistantly across the product width to fit different manufacturing requirements.

The first commercially available system based on this technology, Rometer, was developed by IRM in the mid-1980s [38,39]. It used five laser spots projected onto the surface of the product to be measured. One spot was fixed at the center of the production line, whereas the other four spots were moved to be projected on the sides and on intermediate areas of the product surface based on its width and its position on the production line. This movement was achieved by means of sets of rotatory mirrors. Together with the flatness measurements, two extra signals are provided by this sensor, the symmetrical and the asymmetrical flatness amplitudes.

**Figure 8.**
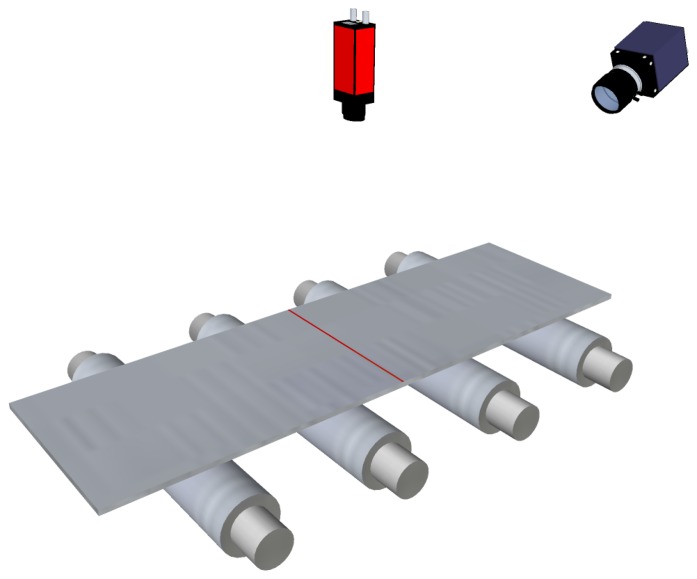
Flatness sensor based on active optical triangulation. A laser stripe is projected across the width of the rolled product and is imaged using a matrix camera.

The evolution of laser technology allowed flatness sensors to use line projectors instead of discrete-point projectors. In these sensors, a matrix camera is used to image the laser stripe projected onto the product surface, as shown in Figure 8. Using a laser stripe projected across the width of the product surface—together with the speed of the product movement while manufacturing—a complete 3D surface reconstruction of the product surface can be obtained. Then, using this reconstruction, the length of several fibers can be calculated to compute flatness measurements. With a line projector, mechanical or optical devices to track the lateral displacements of the product are not required, so the hardware complexity of the system is greatly reduced. This complexity is moved to the software of the sensor. An example of a commercially available flatness sensor based on laser line triangulation is Shapeflex, developed by Shapeline AB [40].

**Figure 9.**
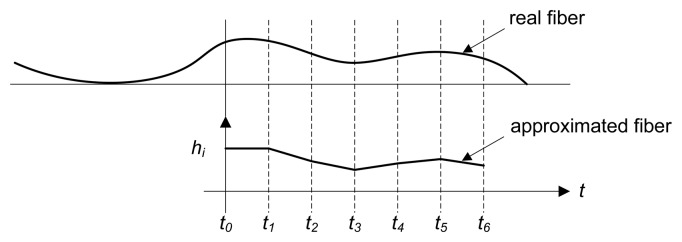
Approximation of the length of a fiber on the surface of a rolled product computed by a triangulation-based flatness sensor.

Flatness sensors based on optical triangulation compute the length of each fiber based on an integration technique that uses the height variations of the fiber between consecutive samples together with the speed of the product movement in the production line. This integration technique is expressed in Equation (6), where *L_j_* is the computed approximation of the length of the fiber, *j*, $h_{i}^{j}$ is the height of the fiber, *j*, in the *i*-th sample, *t_i_* is the time stamp where the sample, *i*, was taken, *v_i_* is the speed of the product movement and *n* is the total amount of samples to compute the length of the fiber. Figure 9 shows how a real fiber is approximated.

(6) $$L_{j}=\sum_{i=1}^{n}\sqrt{{\left(h_{i}^{j}-h_{i-1}^{j}\right)}^{2}+\upsilon_{i}^{2}{\left(t_{i}-t_{i-1}\right)}^{2}}$$

**Figure 10.**
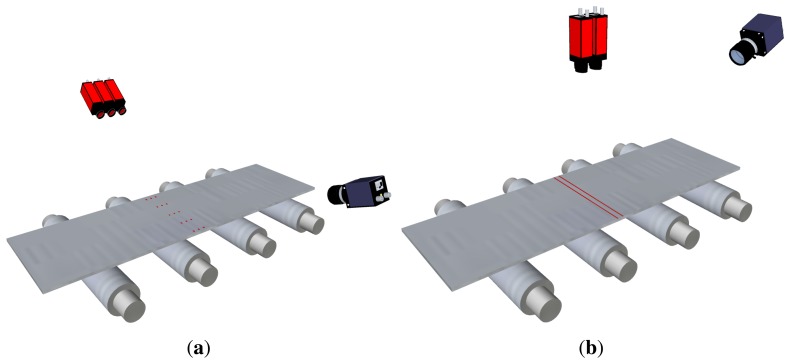
Flatness sensors based on active optical triangulation using multiple laser patterns: (**a**) multi-spot pattern; (**b**) multi-stripe or multi-line pattern.

One of the main issues regarding the active triangulation techniques described above for flatness measurement is how undesired movements of the product, such as bouncing, flapping and rotating, can affect the accuracy of on-line flatness measurements. These movements can be seen as vibrations of the product by the measurement system. In most modern flatness sensors based on active triangulation, two different techniques can be used to avoid introducing the effect of these vibrations in the flatness measurements. On the one hand, multiple arrays of spots or multiple stripes can be projected onto the product surface, as shown in Figure 10. The sensor must be able to remove the vibrations of the product using several consecutive samples of the multiple patterns. On the other hand, mathematical models of the undesired movements of the product can be estimated and used to remove their effect from the topographical information of the product surface using geometrical transformations and filtering before computing the flatness measurements.

An example of a flatness sensor using multiple arrays of laser spots to avoid the effects of undesired movements of the rolled product is an evolved version of the original Rometer. This sensor projects sets of two or three points in the longitudinal direction of the product surface. Figure 10a shows an example using three spots for each longitudinal fiber. The variations observed between these three points are used to remove the effects of undesired movements of the product. In the two-point configuration, each sample contains two simultaneous measurements for each longitudinal fiber of the product. Real waves, or flatness defects, on the product disturb the distance between the two laser spots for the same fiber, whereas vibrations do not modify the relative distance between them. Thus, a consistent topographical representation of the product surface can be computed sampling the product in intervals of the length that matches the distance between the two laser points. In the three-point configuration, each sample contains three simultaneous measurements for each longitudinal fiber of the product. These three measurements feed a system of three equations with three variables: vertical movement, rotation and flatness amplitude. Thus, the effects of undesired movements are avoided. Shapeline also provides its Shapeflex flatness sensor with two laser lines, as shown in Figure 10b, to remove the effects of vibrations from the topographical information of the product surface and, thus, from the flatness measurements.

However, the approaches based on multiple light patterns impose several requirements. First, the patterns must be projected closely on the surface of the rolled product to ensure they suffer the same vibrations. Second, a highly accurate measurement of the speed of the product movement in the production line is required, since between two consecutive samples, some of the points acquired in the first sample under one pattern must be acquired in the second under another pattern. Therefore, the uncertainty in the measured speed introduces noise in the estimation of product vibrations and, thus, in the flatness measurements provided by the system. Furthermore, multi-plane camera focusing requires the lens of the machine vision system to operate with a smaller iris than when imaging only one plane. This is of utmost importance when working in harsh conditions with dark product surfaces or those covered with scale, where the patterns are reflected poorly and big apertures (small F numbers) in the lens are needed. Furthermore, camera calibration is more complex when several patterns are used. Finally, the cost of the systems increases exponentially, since a single-line pattern emitter usually costs more than the image sensor.

As mentioned above, the second option to avoid the effect of undesired movements of the rolled products in flatness measurements consists of removing these movements from the surface reconstruction of the rolled products. Thus, this is a software approach that does not require extra hardware components. A more reliable surface reconstruction without vibration effects can be obtained using geometric transformations when the shape of the product is known previously [41]. Furthermore, a low pass-filter can be used to correct points in the surface reconstruction that do not belong to the product surface due to known physical characteristics of flat-rolled products [42].

#### 3.2.2. Fringe Projection and Moiré Topography

**Figure 11.**
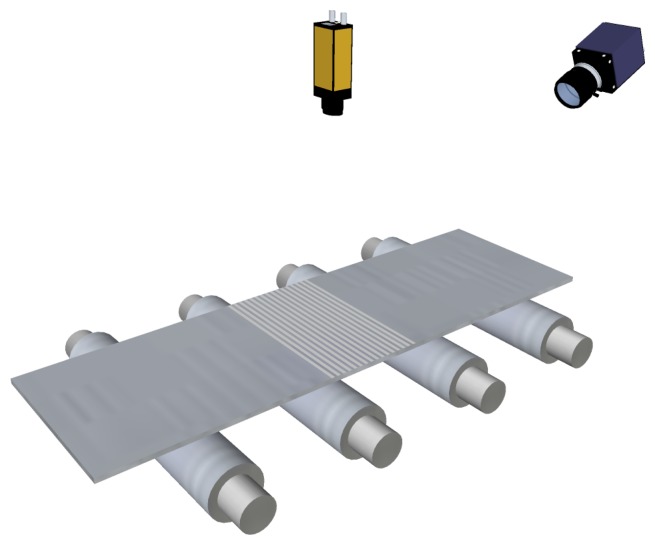
Flatness sensor based on fringe projection.

Fringe projection is an optical technique, where a set of parallel lines, or rectilinear fringes, are projected onto the surface of a product, as shown in Figure 11. The pattern of parallel lines will appear distorted when the surface is not flat and it is observed from an angle different from that projected [43]. This effect is used by the TopPlan flatness sensor developed by BFIand commercialized by IMSMesssysteme to quantitatively provide flatness measurements in steel production lines [44]. The fringe projector of this system adds an aperiodic grid to the rectilinear fringes. This grid has the same density on the material—from the point of view of the camera—when the projection is done at an angle, which simplifies the evaluation of the measurements [45].

Another optical technique that can be used to retrieve the shape of an object is moiré topography [46,47]. This technique uses the effect produced by two gratings overlaid at an angle, known as moiré pattern. Data acquisition and processing is relatively fast, and the contours of specularly reflecting and light scattering surfaces can be retrieved. Moiré topography was first used in automatic flatness inspection in the steel industry in the flatness sensor developed by Paakkari [48] at VTTElectronics. This sensor employs the traditional technique in which a moiré pattern is obtained by projecting a Ronchi grating onto the product surface and comparing it optically with a detection grating.

Vibration effects are not introduced in flatness measurements computed by systems using fringe patterns and moiré topography, since they sample the whole area of the product surface required to compute the flatness measurements simultaneously. That is, the reference length used to compute flatness measurements, *L_ref_*, is shorter than the length of the pattern projected onto the product surface.

#### 3.2.3. Diffused Light Projection

**Figure 12.**
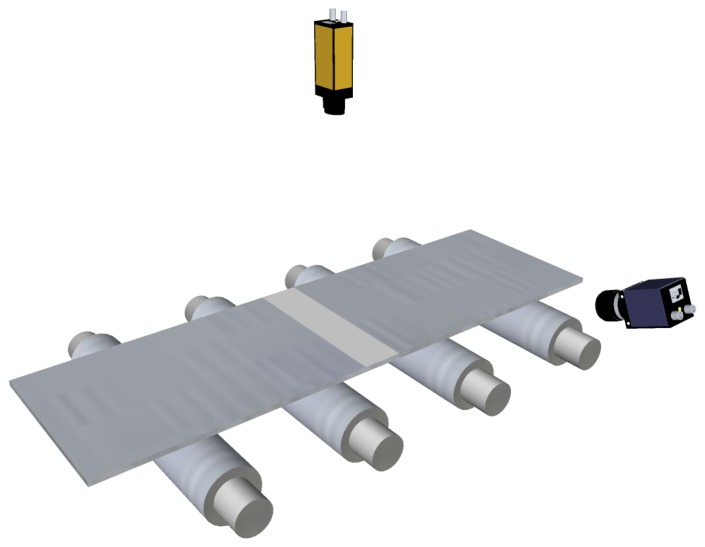
Flatness sensor based on diffuse light projection.

Recently, an optical technique based on diffused light was being used in the steelmaking industry for flatness measurement. This technique requires a very uniform, diffused light ribbon projected onto the surface of the product to be measured. The light ribbon is imaged by a CCD-matrix camera, as shown in Figure 12. When the product is moved along in the production line, changes in the light intensity and light reflection angle are used to compute the shape of the product, unlike triangulation systems, where the shape is measured based on height differences. A commercially available flatness sensor based on this technique, the VIP-08, was developed by Vollmer [49]. In this sensor, standard mercury-vapor lamps are used as light sources to generate an 80 mm-wide ribbon. The light is distributed via light conductor units and mirrors to project the uniform diffuse light ribbon onto the product. Since this sensor evaluates the changes in lighting conditions on the product surface, it requires moving material to provide flatness measurements, similar to the laser scanning methods described above. This flatness sensor is able to track up to 250 longitudinal fibers of the rolled product, and thus, it is able to provide 250 flatness measurements across the product width. This density of measurements lies between that provided by roll-based flatness sensors and that provided by flatness sensors based on laser stripes.

Flatness sensors based on diffuse light projection can be used both in cold and in hot-rolling applications. Furthermore, since height movements do not affect the reflection angles of the light pattern, vibrations of the product do not add noise to the flatness measurements. This technique can only measure manifest flatness defects, similar to the rest of the optical techniques.

### 3.3. Non-Contact, Electromagnetic Flatness Sensors

An alternative, non-contact flatness sensor, SIFLAT, was developed by Siemens in 2005 [50]. This flatness sensor is based on measuring the excitation amplitudes across the width of the product when it is subjected to periodic excitation. These amplitudes are measured using contact-free, electromagnetic distance measurement sensors based on eddy current. Each sensor generates an electromagnetic field, which is proportionally modulated by the distance to the product under inspection. Based on the amplitude measurements, the tension distributions across the product width can be calculated, and then, flatness measurements can be computed. Since the tension profile is computed, latent flatness defects can be identified using this sensor.

Although the principle used by this technique is contactless, systems implementing it require deflection rollers to ensure that no more vertical movement other than that exerted by the system appear in the product. Thus, in the system as a whole, some contact with the product surface is required. Therefore, this technique may not be suitable, as happens with other contact flatness sensors, for high quality surface manufacturing.

### 3.4. Non-Contact, Capacitive Flatness Sensors

Another non-contact technique used to measure the flatness of rolled products in the steelmaking industry is based on the capacitive effect. Distance measurements are retrieved taking the electrical features of the rolled product into account and are used to estimate the length of its fibers [51,52]. Flatness measurements are computed using these measurements. Flatness sensors based on this technique can be used with products featuring bright surfaces, since only electrical characteristics of the rolled product are required to measure the length of the fibers. However, this technique requires the surface of the product to be quite close to the sensor (about 10 cm) and may not be suitable to be installed in some installations and to be used with products with large flatness defects.

## 4. Flatness Sensors for the Steelmaking Industry in Spain

Spain has traditionally been an important steel supplier. Several steel production plants were built in the 20th century, taking advantage of the mining resources of the country and the main maritime ports that allowed importing raw minerals from other countries. In the last decades of the century, a great effort was made to automate the industrial processes and measurement tasks of these plants. Usually, this automation—in continuous evolution—is carried out using both external and internal resources. Steel production plants usually rely on commercial systems and devices to automate process manufacturing and control or quality inspection. On other occasions, steel production plants use solutions specifically designed in their own research and development centers. In the second scenario, given the research orientation of the tasks, many times, these centers become a research partner with some universities.

To the best of our knowledge, no Spanish company has designed or developed commercial flatness sensors for the steelmaking industry. In Spain, one example of a successful partnership between a steel technological center and a university is that between ArcelorMittal's R&D Technological Center, in Avilés, and the University of Oviedo, both in the Principality of Asturias. ArcelorMittal is the world's largest steel producer, and its plant in Asturias is the most important in the country. Several research projects have been collaboratively developed over the years, many of them in the field of measurement automation. Some of these projects resulted in an industrial prototype or a fully-functional system installed in one or more production lines of the plant. When proven successful, these systems are then exported to other plants of the steel producer. The need for ever-better flatness sensors in flat-rolling mills and production lines is a demand of this steel supplier [53]—as it is for all steel suppliers. Thus, several research projects have been collaboratively developed in this area.

Among all the research groups at the University of Oviedo involved in this partnership, two of them are currently carrying out research projects on the automation of flatness measurement of flat-rolled products, both in cold and hot-rolling mills. The first group is with the Department of Computer Science and Engineering. This group has been working in the research, design and development processes of optical flatness sensors based on active optical triangulation for the steelmaking industry since the early 1990s. First, laser triangulation based on discrete points was studied, and a flatness sensor for hot-rolling mills was designed and developed using three laser spots projected onto the surface of the rolled products to be inspected [54–56]. The projection of each laser point was acquired by a 2,048-pixel, CCD-linear camera, and the images provided were processed using a high-speed line scan processor. This system was able to provide height measurements of each longitudinal fiber at 500 Hz, ensuring real-time flatness measurement for high-speed rolling mills and manufacturing lines. The laser points were stationary, so they measured the length of one fiber at the center and one fiber on each side of the rolled product, following the design shown in Figure 7. As described above, flatness sensors based on stationary laser spots cannot properly track the fiber of the rolled product when it moves horizontally on the processing line.

The second flatness sensor designed by this research group was also based on spot-laser triangulation. In this case, five laser spots were projected onto the surface of the product under inspection, and stepping motors were used to both continuously track the longitudinal fiber of the surface and adapt to the width of each rolled product [57–59]. The projection of each laser point was acquired using similar CCD-linear cameras as in the previous system, and the images provided by each camera were processed using a high-speed line scan processor. Height measurements were obtained with sub-pixel accuracy; 0.1 mm in the worst case. This system was proven satisfactory in a hot-rolling mill and influenced the decision to acquire commercial flatness sensors for the plant based on this technique.

The drastic reduction in the costs of laser line projectors led to research tasks on reconstructing the whole surface of rolled products based on laser-stripe triangulation. Using surface reconstruction, metric measurements—like the length of longitudinal fibers—were taken, and flatness measurement was computed. A new flatness sensor was designed and developed by this research group [19,60,61], following the design shown in Figure 8. This system used a 1,280 × 1,024 pixel-resolution, CCD-matrix camera to acquire the projection of the laser stripe projected onto the surface of the rolled product under inspection. A fast, accurate and robust method to extract the laser stripe in harsh industrial environments was designed and used by the system [62]. This method uses an improved split-and-merge approach with Akima splines to fit the laser stripe on the image. Laser-stripe triangulation, together with the computational capabilities of modern computers, allowed this system to be used, both as a flatness sensor and as a width measurement sensor [63]. Like all the optical systems described in Section 3, the flatness sensors designed and developed by this research group are capable of measuring only manifest flatness defects.

As described above, one of the main drawbacks of flatness sensors based on a single laser stripe is the potential errors introduced in the flatness measurements, due to inaccurate surface reconstructions when undesired movements, such as vibrations, appear in the rolled product while manufacturing. Vibrations corrupt the height measurements, causing an erroneous reconstruction of the surface of the rolled product. Thus, flatness measurements are distorted, because the computed lengths of the longitudinal fibers with vibrations differ from the real lengths. As mentioned above, two different solutions can be applied to overcome this issue when using optical laser triangulation. First, multiple lines can be projected and used to accurately reconstruct the surface of the rolled product. Second, the effects of vibrations can be removed from the surface reconstruction before flatness measurements are taken. In the first case, the optical hardware is more expensive, since two or three laser stripes must be generated. This research group developed a new flatness sensor, where the effects of the vibrations introduced in the surface reconstruction of the rolled product are removed by software [64]. In this system, a 2,560 × 2,048 pixel-resolution, CCD-matrix camera is used, and two approaches are proposed for detecting and extracting the line pattern projected on the product surface. One approach was designed focusing on high-speed processing capabilities (up to 200 fps) at lower accuracy (measurement error above 1%) and is based on standard methods for edge detection and linking. The other approach was designed focusing on high accuracy (measurement error below 1%), although incurring higher computational time expenses (the maximum acquisition rate is 40 fps), and is based on more sophisticated methods that employ differential geometry. The procedure to remove or reduce the effects of vibrations is based on a combination of a low-pass filter, to remove high frequency components—associated with vibrations—in the longitudinal fibers, and a geometric transformation compensation using random sample consensus (RANSAC), to remove the remaining low frequency translations and rotations [42].

Apart from the research in optical triangulation for automated flatness sensors in rolling mills, this research group developed, in collaboration with the aforementioned technological center, complementary research tasks for automated flatness measurement. Among them, the most important are: (*i*) on-line and off-line visualization techniques to adequately provide flatness measurements to human operators in rolling mills [65]; (*ii*) compensation for uneven temperature across the width of the rolled product while rolling to avoid flatness defects caused by different temperature profiles [9]; and (*iii*) modeling for flatness optimization in hot-rolling mill processes [17].

The second research group at the University of Oviedo involved in this partnership and working on research tasks related with the automation of flatness measurement in the steelmaking industry is with the Department of Electrical, Electronic, Computer and System Engineering (EECS). This group is focused on the application of the capacitive effect for distance measurement and has designed and developed a low-cost flatness sensor for cold-rolling based on this effect [51,52].

This research group studied the use of capacitive distance measurement based on the electrical features of steel rolled products to retrieve the elongation of several longitudinal fibers of their surface. The distance measurement technique is implemented based on the classical impedance divider circuit, mainly intended to measure floating capacitors. In this design, a capacitor is built up using an electrode and the rolled product itself—the rolled product is considered the electrical ground. The output of this *capacitor* feeds a precision rectifier and a two-stage filter and amplifier that provide a DC signal as a function of the distance between the electrode and the rolled product. A finite element analysis is used to compute the relation between the capacitance and the distance. Finally, taking the distance measurements and the speed of the product movement in the manufacturing line into account, the length of the fibers is computed. The length of each fiber is computed using Equation (6), where $h_{i}^{j}$ represents the distance of the fiber, *j*, to the sensor electrode in the *i*-th sample. This flatness sensor has 24 electrodes of 52 mm each, with a nominal distance from the product surface of 5 cm. In this scenario, the capacitance to be measured is as low as 1 pF. Since this flatness sensor measures the length of the fibers using a non-contact technique, tensile stress across the width of the rolled product is not taken into account, and thus, only manifest flatness defects can be measured. Furthermore, since the sensor needs to be located near the rolled product, it is suitable neither for hot-rolling processes nor for areas in the processing lines where the movement of the product provokes severe undulations.

Another research group involved in this partnership that has developed research tasks in the field of flatness measurement automation is also with the EECS Department. This research group focused its research on measuring the straightness of rolled products in hot-rolling installations to quantitatively evaluate straightness deviations or curvature in the edges of the rolled products, commonly called camber [20]. This research group designed and developed an optical system based on three CCD-matrix cameras to image and track the edges of rolled products during rolling [66]. A three-camera architecture was chosen to image a large region of interest, 20 meters, concatenating three images acquired simultaneously of three adjacent sections of the rolled product. This system differs from all the systems described above, since it does not provide flatness measurements in I-units, but deviations of the rolled product from a perfectly straight edge.

To the best of our knowledge the above are the Spanish research groups working—or that have worked in the past—on flatness measurement automation for the steelmaking industry.

## 5. Conclusions

In this paper, the main technologies and applications for on-line flatness measurement in the steelmaking industry have been reviewed. Most of them can also be used in other industries involving rolling mills or continuous production lines, such as aluminum, copper and paper, to name a few. Encompassed in the special issue, *State-of-the-Art Sensors Technology in Spain 2013*, this paper also reviews the most important flatness prototypes and systems designed and developed for the steelmaking industry in Spain.

Table 1 shows a comparative overview of the main features of the commercially available flatness sensors described in Section 3 and the research-based flatness sensors described in Section 4. As can be seen, most of these sensors are based on non-contact approaches. Contact flatness sensors based on force measurement are the most commonly used flatness sensors in cold-rolling, where the quality of the surface is not so important at this part of the product manufacturing. In downstream processes, where surface quality is important, non-contact flatness devices are preferred. Furthermore, non-contact flatness sensors are used in hot-rolling, where high temperatures may damage roll-based sensors and when flatness of steel plates needs to be measured, since these rolled products are not processed under tension, and thus, tensile stress cannot be measured.

One issue concerning the design of modern flatness sensors is the accuracy of their measurements regardless of the harsh conditions of the rolling mill or the production line where the flat-rolled product is being manufactured. Mechanical-based flatness sensors provide accurate measurements though the tension of the rolled product, while manufacturing is not constant. On the other hand, optical-based sensors implement different approaches to remove the effects of undesired movements of the rolled product while manufacturing. These approaches can be hardware, projecting multiple patterns onto the product surface and using some invariants between these patterns to accurately compute the surface reconstruction of the product, or software, transforming and filtering the height measurements computed from a single pattern onto the surface to avoid noise in the surface reconstruction.

Future flatness sensors for the steelmaking industry will be focused on providing real-time, high-density flatness maps, with more accurate measurements and very low uncertainty—modern on-line flatness sensors provide measurements with a relative uncertainty of 0.1% [67]—in very high-speed rolling mills and production lines.

## Figures and Tables

**Table 1.** Comparison of flatness sensors for flat-rolling or continuous production lines in the steelmaking industry. Most of these sensors can be used in other industries where rolling or continuous production lines are used, such as aluminum, copper and paper.

**Flatness sensor**	**Type**	**Defects**	**Principle**	**Method**	**Sensor**	**Thickness**	**Application**	**W. Resol.**	**L. Resol.**	**Accuracy**
Stressometer® [28]	Contact	Latent	Mechanical	Force measurement	Electromagnetic	Maximum (max.) 2 mm	Cold rolling	260/m	4/rev.	0.5 I-units
BFI roller [30]	Contact	Latent	Mechanical	Force measurement	Piezoelectric(1)	–(2)	Cold rolling	n/a	6/rev.(1)	–(2)
CLECIM [32]	Contact	Latent	Mechanical	Force measurement	n/a	n/a	Cold, hot rolling	n/a	n/a	n/a
Rometer [39]	Non-contact	Manifest	Optical	Spot laser triangulation	CCD linear/matrix	No max.	Cold, hot rolling	3 and 5	n/a	n/a
Shapeflex [40]	Non-contact	Manifest	Optical	Laser triangulation	CCD matrix	No max.	Cold, hot rolling	n/a	n/a	0.1 mm(3)
TopPlan [44]	Non-contact	Manifest	Optical	Fringe projection	CCD matrix	No max.	Cold, hot rolling	n/a	n/a	n/a
*Paakkari* [48]	Non-contact	Manifest	Optical	Moire topography	CCD matrix	No max.	Cold, hot rolling	n/a	n/a	0.1 mm(3)
VIP-08 [49]	Non-contact	Manifest	Optical	Diffuse light projection	CCD matrix	No max.	Cold, hot rolling	250	50 Hz	0.01 I-units
SIFLAT [50]	Non-contact	Latent	Mechanical	Distance measurement	Electromagnetic	Max. 4 mm	Cold rolling	n/a	10 Hz	n/a
*Garcίa* [56]	Non-contact	Manifest	Optical	Spot laser triangulation	CCD linear	No max.	Cold, hot rolling	3	500 Hz	n/a
*Garcίa* [59]	Non-contact	Manifest	Optical	Spot laser triangulation	CCD linear	No max.	Cold, hot rolling	5	500 Hz	0.1 I-units
*Lopera* [51]	Non-contact	Manifest	Capacitive	Distance measurement	Capacitive array	No max.	Cold rolling	24	5 cm	n/a
*Molleda* [19,64]	Non-contact	Manifest	Optical	Laser triangulation	CCD matrix	No max.	Cold, hot rolling	200(4)	100 Hz	0.15 I-units

W. resol.: Width resolution, *i.e.*, number of flatness measurements provided across the width of the rolled product; L. resol.: Longitudinal resolution, *i.e.*, number of flatness measurements (contact sensors) or height measurements (non-contact sensors) provided along the rolled product or per time unit;

(1) In modern implementations;

(2) Manufacturer-dependent;

(3) Accuracy of height measurements in the product surface (accuracy in I-units is not provided);

(4) User configurable.
